# A Novel Technique for the Removal of Elastic Intramedullary Nail in Pediatric Long Bones: A Technical Note

**DOI:** 10.7759/cureus.9717

**Published:** 2020-08-13

**Authors:** Vinay K Gautam, Ashish S Ranade, Mahesh Mone, Gauri A Oka

**Affiliations:** 1 Orthopaedic Surgery, Deenanath Mangeshkar Hospital and Research Centre, Pune, IND; 2 Orthopaedics, Blooming Buds Centre for Pediatric Orthopaedics, Deenanath Mangeshkar Hospital and Research Centre, Pune, IND; 3 Research, Deenanath Mangeshkar Hospital and Research Centre, Pune, IND

**Keywords:** nail removal, elastic stable intramedullary nailing, pediatric long bone fracture

## Abstract

Elastic stable intramedullary nails have been commonly used to treat unstable long bone fractures in children. The nail tip at the insertion site can cause problems. The nail tip should be of optimal length as a prominent nail tip or a short nail tip, or both, may cause different sets of problems. If the nail tip is short, nail removal after fracture union can be difficult and may pose challenges. A short nail tip may lead to difficulty in nail removal, longer duration of surgery, and need for special equipment for extracting the nail. Few techniques have been suggested in the past for removing elastic nail with the short tip, but all these techniques need special instruments. We describe a surgical technique using a metallic suction cannula to aid elastic nail removal. This method utilizes an easily available instrument in the operating room and does not need any special equipment.

## Introduction

Elastic stable intramedullary nailing (ESIN) is a commonly used modality for surgical stabilization of long-bone fractures in children. It is widely used for treating unstable fractures of the radius, ulna, femur, and, occasionally, the tibia and the humerus. It has also been used to treat pathological fractures of the long bones in children. There are several advantages of ESIN. ESIN offers closed fracture fixation without opening the fracture site, three-point stability, and maintenance of length and rotation in transverse, short-oblique fractures. Being a load-sharing implant, it allows for early mobilization of the extremity. Routinely, elastic stable intramedullary nail is removed after fracture union. Sometimes, early removal or repositioning is necessary for a prominent painful tip of the nail. To facilitate removal, the tip of the elastic stable intramedullary nail if left proud at the entry site can cause pain and irritation. Nail tip prominence can also lead to skin breakdown, tendon rupture, and need for early implant removal. However, if the nail tip is impacted in the bone, then the removal is difficult. This difficult nail removal cannot be always predicted on pre-operative radiographs. Removal of the intramedullary nail after fracture union can be difficult, and there are reports of incomplete removal [1-3]. Few techniques have been described for the removal of the elastic nail [4-6].

We describe a technique using a metallic suction cannula to remove the elastic nail when the tip of the nail is short and cannot be easily held with a plier or similar instrument.

## Technical report

**Figure 1:**
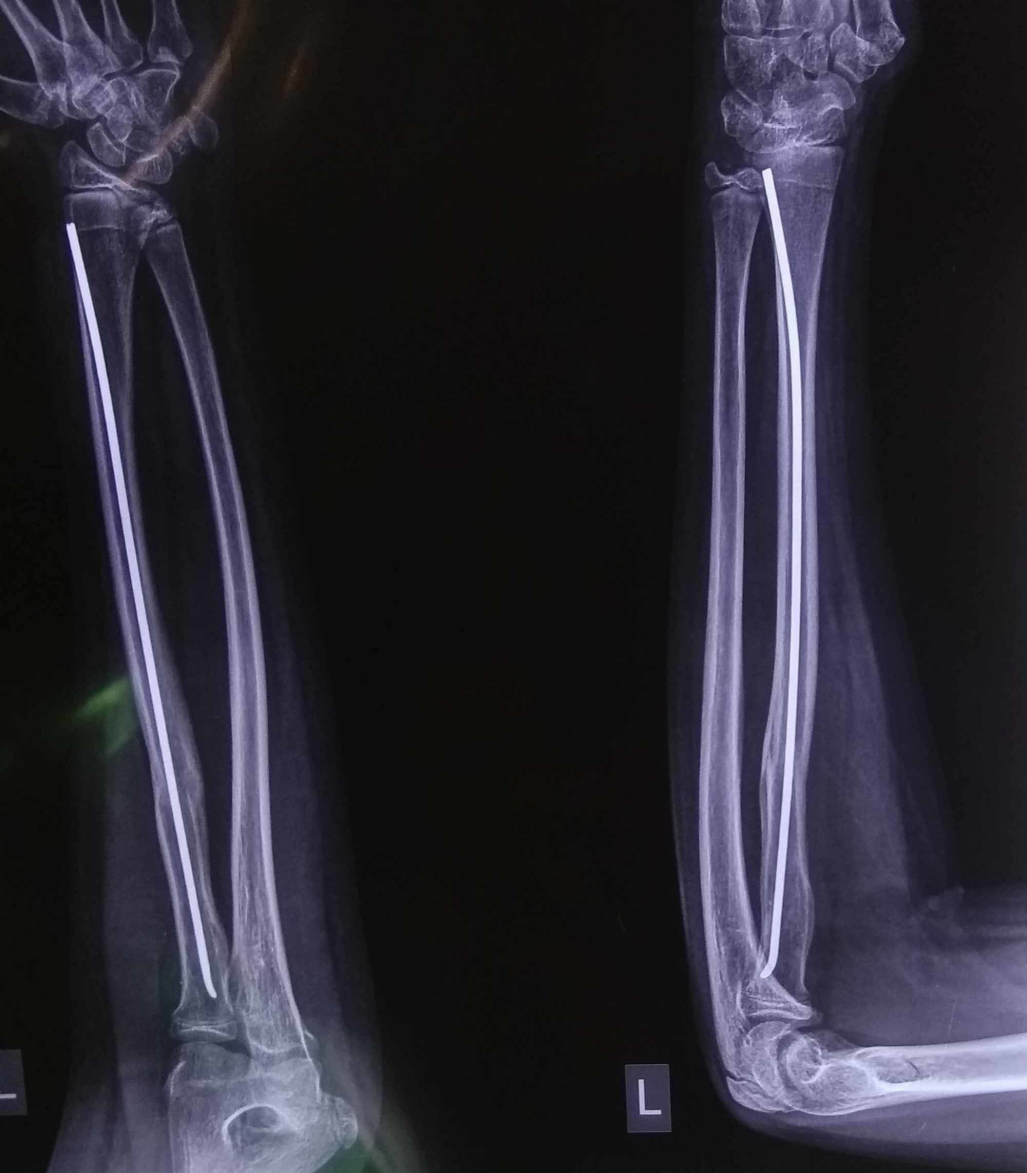
Anteroposterior and lateral radiograph of the united radial fracture with in situ elastic nail.

The patient, a 12-year-old boy, was operated for isolated unstable radial shaft fracture 10 months ago with ESIN using a 2-mm nail. Clinically and radiologically, the fracture had united and the nail tip was not prominent, as seen in Figure 1.

**Figure 2:**
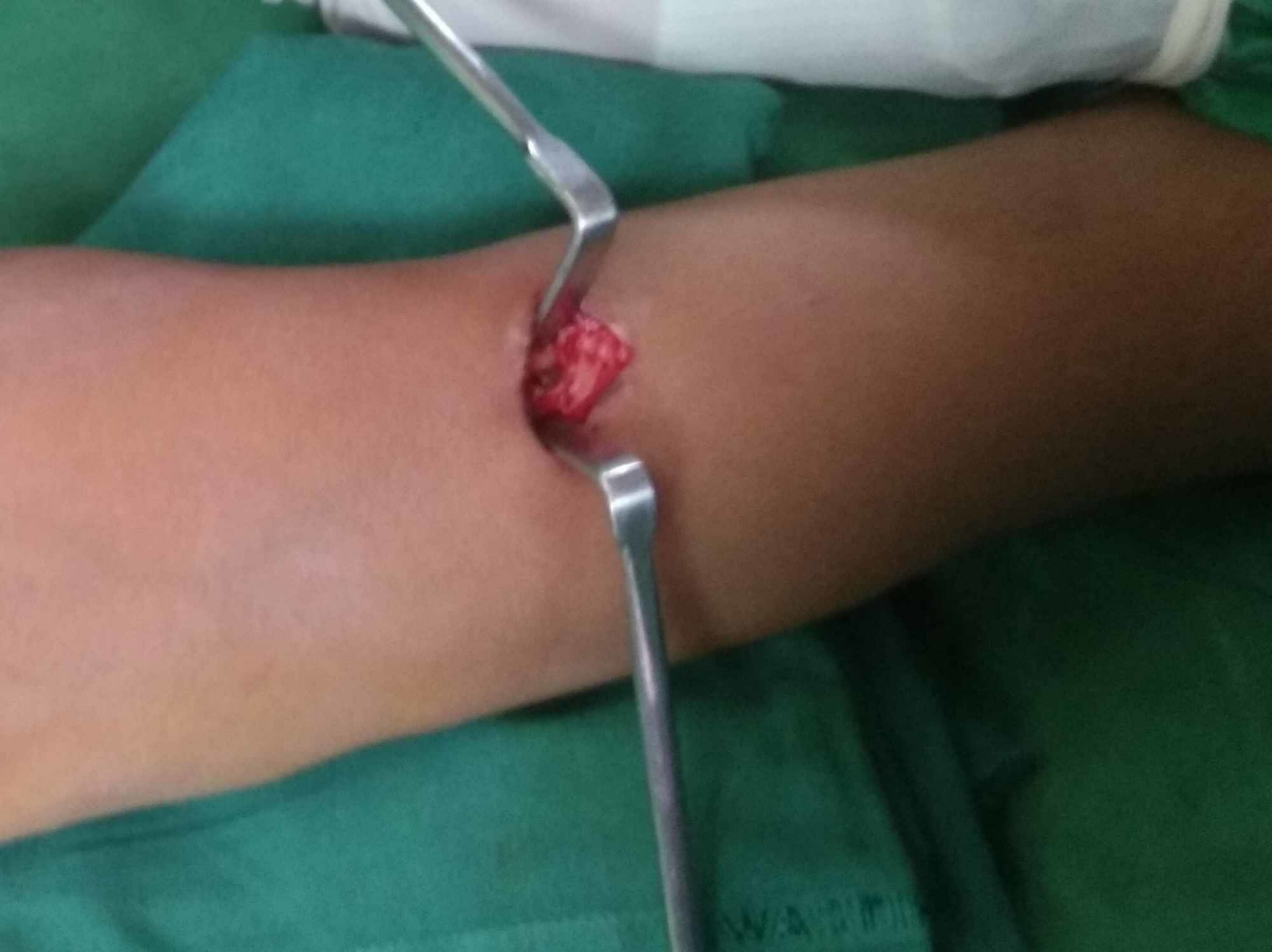
Photograph showing barely visible nail tip, which was not possible to hold with pliers.

Surgery was performed under general anesthesia, with the affected forearm placed on a radiolucent side table. Tourniquet was inflated at 180 mm of Hg pressure. The incision was made on the previous surgical scar. The length of the incision was 2 cm. The nail diameter was 2 mm. Blunt dissection was made to spread the soft tissues, and the nail tip was exposed. It was not possible to grip the tip of the nail using a needle-nosed plier (Figure 2).

**Figure 3:**
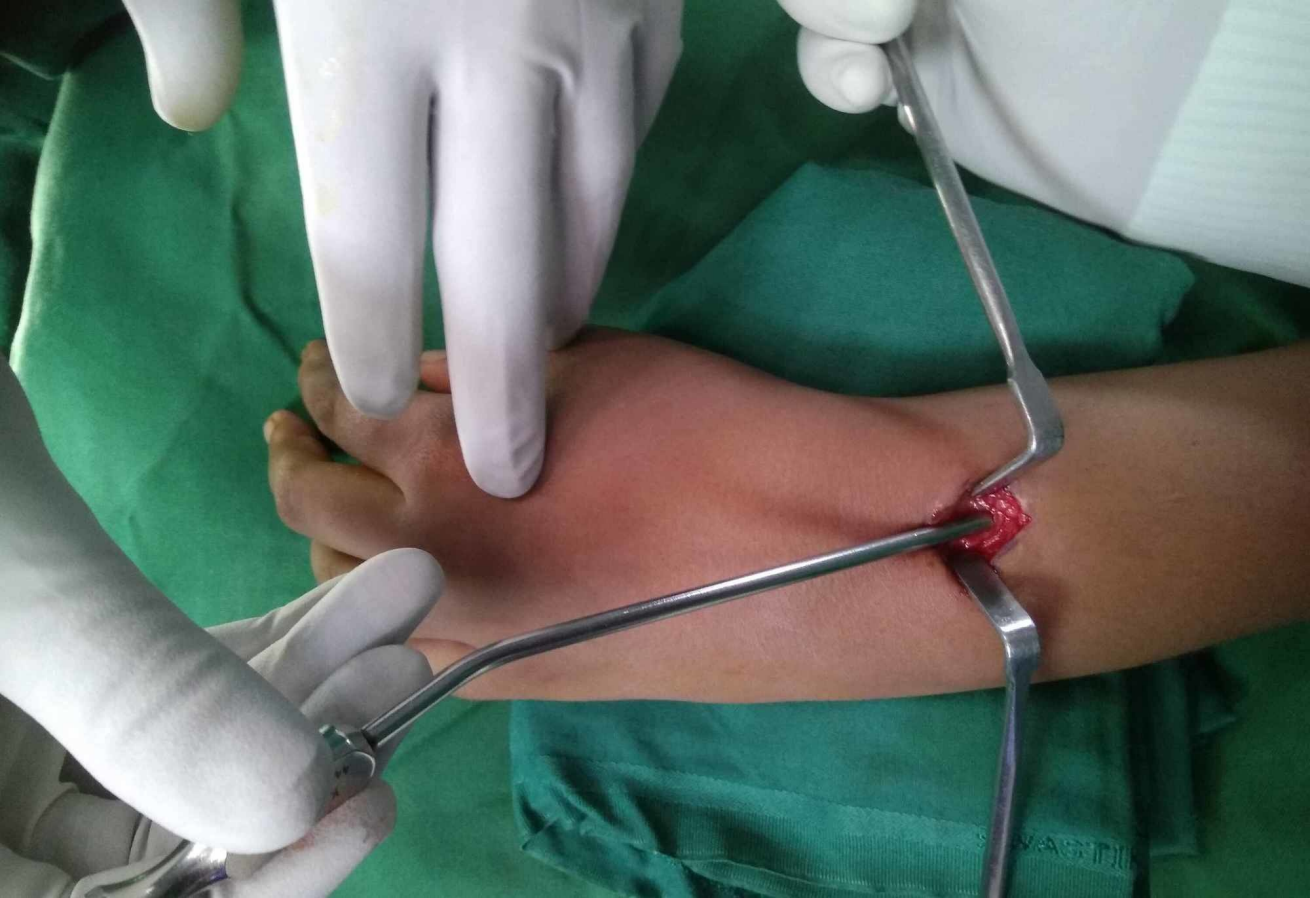
Photograph showing passage of a metal suction cannula over the nail tip.

A metal suction cannula of 3 mm in diameter was slid over the nail tip. The nail diameter was 2 mm. the metal suction cannula was made by a local instrument company. The cannula was pushed as deep as possible (Figure 3).

**Figure 4:**
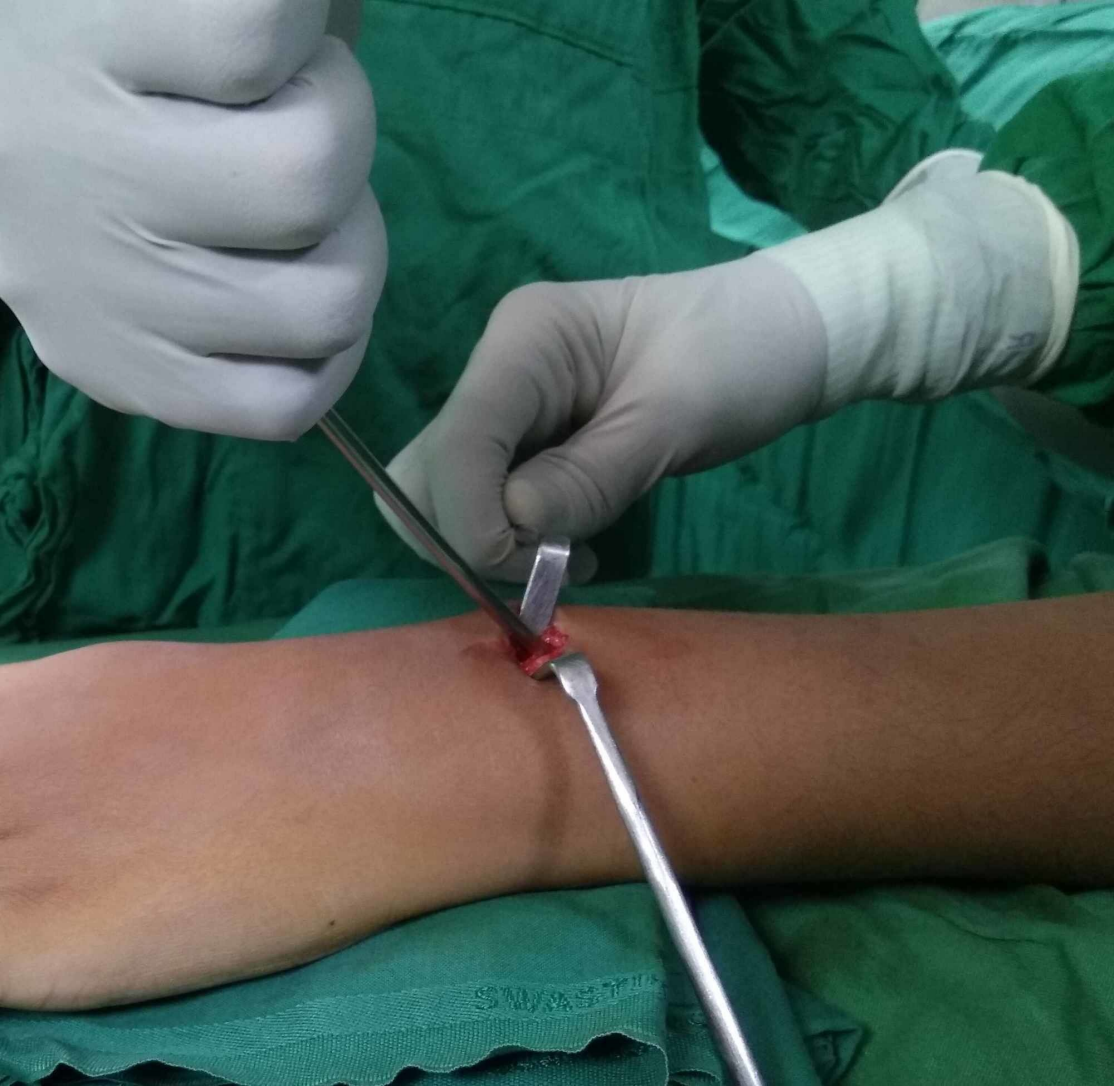
Photograph showing bending of the nail tip using the suction cannula.

After securing the cannula tip over the nail tip, the suction cannula was carefully bent so as to bend the nail tip. The tip was bent to approximately 30 degrees (Figure 4).

**Figure 5:**
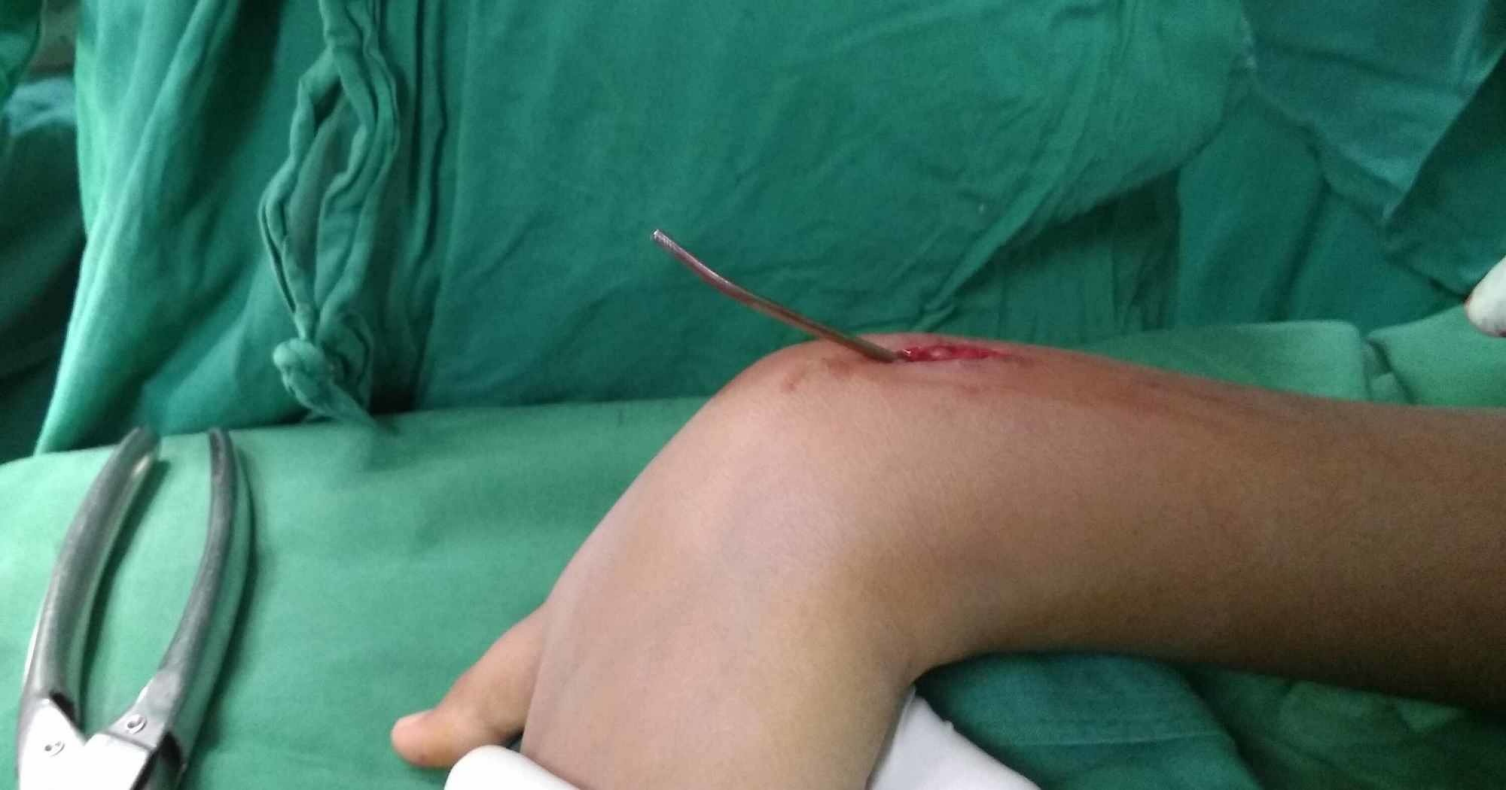
Photograph demonstrating the bent tip and nail that was extracted using pliers.

**Figure 6:**
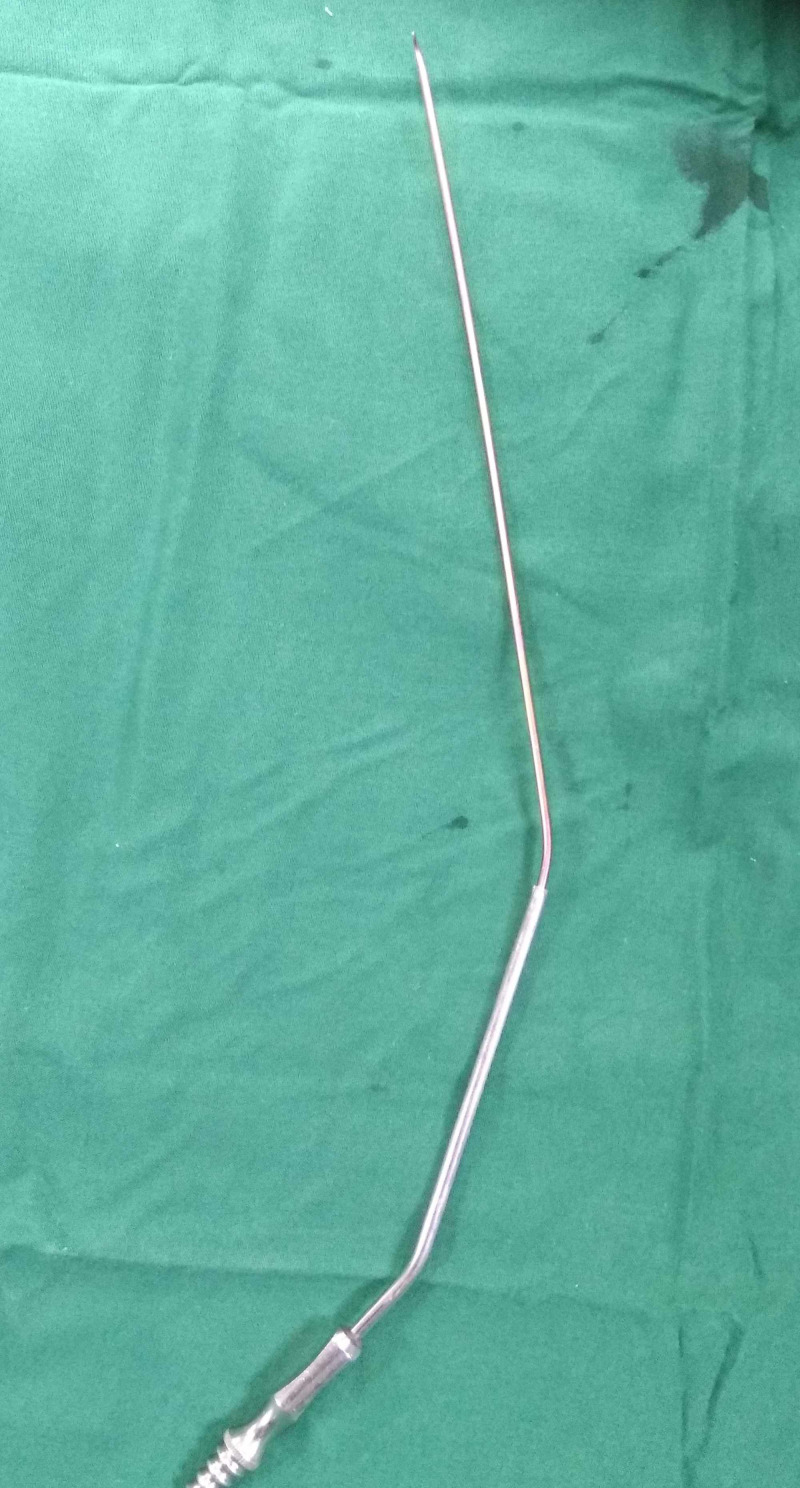
Photograph showing passage of the suction cannula over the bent end of the nail.

At this point, the cannula was removed, and the plier was used to grab the nail tip and the nail was extracted using a combination of longitudinal traction and rotatory movements (Figures 5, 6).

The surgical time was 10 minutes. The wound was closed in a standard fashion. The patient made an uneventful recovery after the surgery.

## Discussion

Removal of the elastic intramedullary nail can pose some challenges and might turn out to be a difficult surgery. Several factors can lead to difficulties while removing an elastic stable intramedullary nail. These factors include a short nail tip, the nail tip being very close to the bone surface, and intraosseous migration of the nail tip. Because of these issues, the plier or the holding instrument may not be able to grip the tip of the nail. In this situation, the bone around the nail tip is removed using an osteotome or a drill. This creates sufficient space for the pliers or vice grip to hold the nail tip. To accommodate the plier, it might be necessary to increase the length of the incision leading to a longer scar.

There are reports describing complications of ESIN removal in a healed fracture in the form of unsuccessful nail removal [1-3]. A few strategies have been described in the literature for the removal of the elastic intramedullary nail. These techniques include the use of a bone chisel around of tip of the nail [5], the use of a hollow reamer and [6], and the use of a broken screw removal device [4]. Lascombes has described the use of the chisel followed by pliers [5]. This typically involves making a larger incision to accommodate the tip of the pliers and also involves the removal of bone using an osteotome or a chisel. McDonnell et al. have described a technique using a cannulated reamer [6]. For this technique, availability of a cannulated reamer in the operating room is a prerequisite. Ferry and Dahners described the use of an extraction bolt from the Synthes screw removal set (Synthes 105.971, Paoli, PA, USA) [4]. The authors also described the use of hollow reamers from the screw removal set to overdrill the cut end of the nail. However, these devices could be expensive and might not be easily available. Our technique requires a metallic tip suction cannula. The removal of nail is performed with minimal soft tissue dissection. The size of the suction cannula can vary according to the diameter of the nail. The suction cannula is locally made, and the typical length is 25 cm. The suction cannula is available in two sizes: 3- and 5-mm internal diameter. We have not encountered any problems with using a suction cannula, which is made up of stainless steel, of any company. The compatibility of the suction cannula with the diameter of the elastic nail is an important consideration. The suction cannula that we use is usually slightly oversized than the nail diameter so that it fits easily up to nails of 3.5-mm diameter. After the nail removal, the suction cannula was not discarded and we used it for subsequent surgeries after sterilization. It is not a disposable cannula and hence does not add additional cost. No special instrument such as the cannulated reamer is required, which may not be available everywhere. Also, our technique does not require nibbling or osteotomy of the bone to expose the tip of the nail.

While using this technique, we did not encounter any complications. Our team has been practicing this technique since January 2017, with excellent results. We have used the suction cannula technique for the removal of 38 elastic nails from 20 patients. In total, we have removed 15 nails from radius, 15 from the ulna, and 8 from the femur. There were 4 patients with femur fracture and 14 patients with both radius and ulna fracture, and 1 patient with isolated radius and 1 with isolated ulna fracture, both treated with ESIN. There were 14 males and 6 females. The average age at the time of implant removal was 14.1 years (range: 8.2-15.4 years). All implants were removed after a mean of 12 months (range: 10-24 months) after fracture fixation. The nail size ranged from 2 mm to 3.5 mm, and the size of the suction cannula was 3 mm or 5 mm of internal diameter. Theoretically, the tip of the nail can break off during bending maneuver; however, in our experience, we have not encountered this situation. Also, the tip of the cannula may bend while trying to bend the nail. However, we have not encountered this situation. We acknowledge the limitation of this technique, that is, it cannot be used when the nail tip has migrated inside the bone. Also, this technique will not work if the nail tip is buried under new bone, when the implant removal is performed several years after surgery. Major drilling or bone removal was not required in our technique because the exposed tip of the elastic nail is sufficient to pass a metallic suction cannula over the tip. The use of the suction cannula described in this technique is a very practical solution that does not require any specialized instrument.

## Conclusions

This method is easy to use, is inexpensive as the cannula is reusable, and does not require new or additional equipment. The technique does not require a lengthy procedure and is found to be very easy to use for surgeons of different experience levels. We recommend this easy technique for the removal of elastic intramedullary nails. The technique we have described is cheap, effective, and can be performed by any surgeon anywhere.
